# Predictive value of combined estimated plasma volume status and neutrophil-to-lymphocyte ratio for cardiovascular mortality in patients with acute myocardial infarction with heart failure

**DOI:** 10.3389/fcvm.2026.1948520

**Published:** 2026-09-28

**Authors:** Caizhi Dai, Xiaomin Chen, Xiuting Chen, Guoli Lin

**Affiliations:** Affiliated Hospital of Putian University, Putian, China

**Keywords:** acute myocardial infarction, estimated plasma volume status, heart failure, neutrophil-to-lymphocyte ratio, risk stratification model

## Abstract

**Objective:**

Estimated plasma volume status (ePVS) and neutrophil-to-lymphocyte ratio (NLR) are novel biomarkers reflecting hemodynamic load and systemic inflammatory immune status, respectively. This study aimed to explore the prognostic value of combined ePVS and NLR for cardiovascular mortality in patients with heart failure (HF) complicating acute myocardial infarction (AMI).

**Methods:**

This retrospective observational study consecutively enrolled patients diagnosed with AMI complicated by HF at Putian University Affiliated Hospital from January 2020 to June 2024. Admission hemoglobin and hematocrit levels were used to calculate ePVS, while routine blood test parameters were applied to determine NLR. The primary endpoint was cardiovascular mortality within two years after AMI. Cox proportional hazards regression models were constructed to analyze the associations of ePVS and NLR with clinical endpoints. The predictive efficacy of single and combined ePVS and NLR was comprehensively evaluated using the area under the receiver operating characteristic curve (AUC), net reclassification improvement (NRI), and integrated discrimination improvement (IDI).

**Results:**

A total of 2046 patients were ultimately included. During a mean follow-up of 819 days, endpoint events occurred in 14.90% of the study population. Univariate Cox regression analysis demonstrated that ePVS served as a strong risk factor for cardiovascular mortality (HR = 1.571, 95% CI: 1.502–1.642, *P* < 0.001). After adjusting for age, diabetes mellitus, hypertension, atrial fibrillation, and NLR, both ePVS (HR = 1.436, 95% CI: 1.370–1.505, *P* < 0.001) and NLR (HR = 1.330, 95% CI: 1.258–1.406, *P* < 0.001) remained independent predictors of cardiovascular mortality. The combined predictive model integrating ePVS and NLR exhibited significantly superior predictive performance compared with either biomarker alone (AUC: 0.794 for ePVS vs. 0.891 for ePVS + NLR, *P* < 0.001). Both NRI and IDI analyses further verified the significant improvement in predictive accuracy of the combined model (all *P* < 0.01).

**Conclusion:**

ePVS and NLR represent valuable prognostic biomarkers for cardiovascular mortality in AMI patients. The combination of ePVS and NLR significantly improves predictive performance and facilitates precise risk stratification.

## Introduction

Acute myocardial infarction (AMI) is a prevalent cardiovascular disease. Despite remarkable advances in revascularization therapy that have reduced in-hospital mortality among AMI patients, several studies have reported an unacceptably high post-discharge mortality rate, which is largely attributed to heart failure (HF) secondary to AMI (1). The incidence of in-hospital HF ranges from 14% to 36% in patients with AMI, and approximately 25% of AMI survivors develop HF within one year after discharge (2). Accordingly, although the success rate of emergency treatment for AMI has been greatly improved, the incidence of post-AMI HF has not decreased substantially, resulting in elevated readmission and mortality rates as well as poor long-term prognosis. Hence, early and accurate identification of high-risk individuals for post-AMI HF is essential to optimize secondary prevention strategies.

Post-AMI HF arises from a complex pathophysiological process involving mechanical dysfunction (3), neurohormonal activation (4), cardiac inflammatory, immune isturbances (5), alterations in ventricular geometry and cardiac function (6), ischemia/reperfusion injury (7), abnormal energy metabolism (8) and genetic factors (9). Among these mechanisms, systemic inflammation and excessive volume overload are recognized as two core drivers of disease progression and adverse clinical outcomes. On the one hand, the robust inflammatory response triggered by AMI exacerbates myocardial injury and promotes adverse ventricular remodeling, and persistent low-grade inflammation keeps HF patients at sustained high risk (10). On the other hand, increased volume overload caused by excessive neuroendocrine activation and impaired renal function not only precipitates decompensated HF but also has been validated as an independent prognostic factor regardless of left ventricular ejection fraction (11). Nevertheless, the combined assessment of these two key pathophysiological dimensions remains inadequate in routine clinical practice, and integrated, objective and readily available predictive tools are still lacking.

Estimated plasma volume status (ePVS), calculated using hemoglobin and hematocrit according to the formula proposed by Duarte et al., is a simple and accessible biomarker that reliably reflects actual systemic volume status. Accumulated evidence has demonstrated that ePVS is closely associated with readmission and mortality risks in HF patients (8). The neutrophil-to-lymphocyte ratio (NLR) is a comprehensive inflammatory indicator that integrates innate immunity (neutrophils) and adaptive immunity (lymphocytes). Elevated NLR has been proven to independently predict adverse cardiovascular events in patients with AMI (12).

Notably, while ePVS and NLR provide distinct prognostic information from the perspectives of volume overload and inflammation respectively, few studies have combined these two biomarkers for risk assessment in patients with post-AMI HF. Therefore, this retrospective cohort study aimed to systematically analyze the associations of ePVS and NLR with long-term mortality in patients with AMI complicated by HF. We further explored the independent predictive value and optimal cut-off values of combined ePVS and NLR for long-term mortality, and established a simple and efficient combined predictive model for this patient population.

## Materials and methods

### Study design and study population

This was a single-center, retrospective cohort study. We consecutively enrolled patients diagnosed with acute myocardial infarction complicated by heart failure between January 2020 and June 2024. The inclusion criteria were as follows: (1) age ≥ 18 years; (2) diagnosis of AMI and heart failure according to the universal definition jointly issued by the European Society of Cardiology (ESC), American College of Cardiology (ACC) and American Heart Association (AHA). Heart failure complicating acute myocardial infarction in-hospital was defined as new-onset heart failure at index admission. Diagnosis was based on composite criteria: clinical signs of pulmonary congestion, Killip class ≥ II, left ventricular ejection fraction (LVEF) < 50%, and elevated NT-proBNP. Patients with documented pre-existing chronic heart failure prior to the index AMI event were identified and distinguished in the dataset. Exclusion criteria included: (1) severe hepatic or renal dysfunction; (2) concomitant severe infection, hematological diseases, autoimmune disorders or advanced malignant tumors; (3) prior use of glucocorticoids or immunosuppressants before admission; (4) incomplete key clinical data.

This study was conducted in accordance with the principles of the Declaration of Helsinki and approved by the Ethics Committee of Affiliated Hospital of Putian University. Given the retrospective nature of this study and full protection of patient privacy, the requirement for written informed consent was waived.

### Data collection and definitions

Baseline demographic characteristics, vital signs, medical history and medication history were extracted from the electronic medical record system. Admission blood samples were obtained at emergency-department presentation on arrival for AMI, prior to revascularization procedures. Routine hematological and biochemical tests were performed in the clinical laboratory of our hospital. Transthoracic echocardiography was conducted by experienced sonographers, and left ventricular ejection fraction (LVEF) was measured using the biplane Simpson's method.

### Calculation of exposure variables

ePVS was calculated using the formula:

ePVS=[100×(1−hematocrit)]/hemoglobin(g/dL)

NLR was calculated as:

$$\begin{array}{r} \mathrm{NLR}=\mathrm{absolute}\;\mathrm{neutrophil}\;\mathrm{count}(\times 10^{9}/L)/\\ \mathrm{absolute}\;\mathrm{lymphocyte}\;\mathrm{count}\;(\times 10^{9}/L) \end{array}$$

### Clinical endpoint and follow-up

The primary clinical endpoint was cardiovascular mortality. Follow-up was performed via outpatient re-examinations, telephone interviews and review of hospital readmission records by researchers who were blinded to the baseline laboratory results. A total of 305 cardiovascular-mortality events were recorded in the cohort. No composite MACE endpoint was analysed in the present study.

### Statistical analysis

Continuous variables were presented as mean ± standard deviation or median (interquartile range) based on normality testing. Intergroup comparisons were performed using the independent *t*-test or Mann–Whitney U test. Categorical variables were expressed as counts (percentages) and compared using the chi-square test. Patients were stratified according to the optimal cut-off values or clinically recognized thresholds of ePVS and NLR. The area under the receiver operating characteristic curve (AUC) was calculated using R software.Kaplan–Meier survival curves were plotted, and the log-rank test was used for between-group comparisons. Univariate and multivariate Cox proportional hazards regression models were applied to evaluate the associations of ePVS and NLR with cardiovascular mortality. Results were reported as hazard ratios (HR) with 95% confidence intervals (CI). Three Cox proportional-hazards regression models were constructed: Model 1 was the unadjusted model containing only ePVS. Model 2 was adjusted for age, diabetes mellitus, hypertension, atrial fibrillation, and NLR. Model 3 was further adjusted for STEMI, peak troponin-I, left-ventricular ejection fraction, Nt-pro-BNP, blood urea nitrogen, and uric acid. Sex was not retained in multivariable models given the absence of independent predictive effect in our study population. Time-dependent receiver operating characteristic (ROC) analysis tailored for censored survival data was performed at a predefined 720-day landmark to evaluate the discriminatory ability and incremental prognostic value of ePVS and NLR. Changes in the area under the curve (ΔAUC) with 95% confidence intervals (CIs) were calculated to quantify predictive improvement. Internal validation of the fully adjusted prognostic model was conducted using 500 bootstrap resamples to obtain the optimism-corrected C-index. Model calibration was assessed using calibration plots with calibration intercept and slope calculated to quantify the agreement between predicted and observed event risks. Decision curve analysis (DCA) was performed at the 720-day landmark to evaluate the net clinical benefit of the predictive model across different threshold probabilities. Fine-Gray competing-risk regression was applied to account for non-cardiovascular death as a competing event for cardiovascular mortality. Cause of death was adjudicated systematically by reviewing inpatient medical records, discharge summaries, and standardized follow-up information. Cardiovascular death was defined according to standardized clinical endpoint criteria. The proportional hazards (PH) assumption was verified using Schoenfeld residual tests. Pearson correlation analysis and variance inflation factor (VIF) were calculated to evaluate the correlation and multicollinearity between ePVS and NLR. All survival analyses were performed on covariates-complete datasets. Net reclassification improvement (NRI) and integrated discrimination improvement (IDI) were calculated to quantify the overall net improvement in sensitivity and specificity of the combined predictive model. Baseline characteristics, univariate Cox regression, and descriptive statistics were performed using SPSS version 26.0. Time-dependent ROC analyses, NRI, IDI, nested Cox models and data visualization were implemented in R (version 4.4.2) with packages “survival”, “rms”, “survAUC” and “ggplot2”. A two-tailed *P* value < 0.05 was considered statistically significant.

## Results

### Baseline clinical characteristics

We consecutively enrolled patients diagnosed with acute myocardial infarction complicated by heart failure who were admitted to Affiliated Hospital of Putian University between January 2020 and June 2024. Mean follow-up was 819 days (25th–75th percentile: 401–1122 days). All endpoint analyses were censored at 730-days after admission. Patients with active infection, hematological diseases, autoimmune disorders, advanced malignancy, or prior immunosuppressant/glucocorticoid treatment were strictly excluded during initial screening to avoid potential interference with systemic inflammatory indices including NLR. A total of 2,250 patients were initially screened. Among them, 126 patients were lost to follow-up and 78 were excluded due to incomplete data. Ultimately, 2,046 participants were included in the final analysis. According to the occurrence of cardiovascular mortality during follow-up, all enrolled patients were divided into the cardiovascular mortality group (*n* = 305) and the non-cardiovascular mortality group (*n* = 1,741) (Supplementary Figure S1). Baseline demographics, comorbidities, laboratory parameters and cardiac function indicators were compared between the two groups (Table 1). For categorical variables, the cardiovascular mortality group had a significantly higher prevalence of diabetes mellitus (43.9% vs. 33.9%, *P* = 0.001), hypertension (69.5% vs. 60.1%, *P* = 0.002) and atrial fibrillation (15.4% vs. 5.7%, *P* < 0.001). No significant differences were observed between the two groups in terms of smoking history, ST-elevation myocardial infarction and lesions in different coronary artery branches (*P* > 0.05). With regard to continuous variables, patients in the cardiovascular mortality group were older (67.19 ± 11.69 years vs. 62.36 ± 11.95 years, *P* < 0.001) and had lower diastolic blood pressure (74.33 ± 15.56 mmHg vs. 76.43 ± 13.40 mmHg, *P* = 0.030). The cardiovascular mortality group presented with significantly elevated levels of white blood cell count, red blood cell distribution width, neutrophil count, blood urea nitrogen, uric acid, serum glucose, serum potassium,ePVS and NLR (*P* < 0.05). In contrast, hemoglobin, lymphocyte count and LVEF were markedly decreased in the cardiovascular mortality group (*P* < 0.001). Additionally, the level of N-terminal pro-B-type natriuretic peptide (NT-proBNP) was substantially higher in the cardiovascular mortality group (11,019.36 ± 10,049.44 pg/mL vs. 3,842.96 ± 2,875.82 pg/mL, *P* < 0.001). The peak troponin-I value in patients with cardiovascular death was significantly increased. Systolic blood pressure, total cholesterol, total bilirubin, serum sodium and glycated hemoglobin were comparable between the two groups (*P* > 0.05).

**Table 1:** Baseline characteristics of the subjects included in the study.

Variable	Cardiovascular mortality group (*n* = 305)	Non-cardiovascular mortality group (*n* = 1,741)	*P*-value
Smoking, *n* (%)	138 (50.2)	872 (52.2)	0.525
Diabetes mellitus, *n* (%)	134 (43.9)	590 (33.9)	0.001
Male, *n* (%)	240 (78.7)	1,428 (82.1)	0.173
Hypertension, *n* (%)	212 (69.5)	1,046 (60.1)	0.002
Atrial fibrillation, *n* (%)	47 (15.4)	99 (5.7)	<0.001
ST-elevation myocardial infarction, *n* (%)	165 (54.5)	913 (52.6)	0.555
Left main coronary artery disease, *n* (%)	15 (5.0)	50 (2.9)	0.056
Left anterior descending artery disease, *n* (%)	177 (58.0)	965 (55.4)	0.398
Left circumflex artery disease, *n* (%)	80 (26.2)	442 (25.4)	0.756
Right coronary artery disease, *n* (%)	99 (32.5)	630 (36.2)	0.210
Age (years)	67.19 ± 11.69	62.36 ± 11.95	<0.001
Systolic blood pressure (mmHg)	129.29 ± 25.30	128.87 ± 20.86	0.793
Diastolic blood pressure (mmHg)	74.33 ± 15.56	76.43 ± 13.40	0.030
White blood cell count (×10⁹/L)	10.83 ± 4.87	9.95 ± 3.79	0.006
Red blood cell distribution width (%)	13.06 ± 1.00	12.83 ± 0.90	0.001
Neutrophil count (×10⁹/L)	9.24 ± 4.56	7.56 ± 3.68	<0.001
Lymphocyte count (×10⁹/L)	1.13 ± 0.45	1.67 ± 0.69	<0.001
Hemoglobin (g/L)	122.82 ± 13.67	149.08 ± 13.65	<0.001
Blood urea nitrogen (mmol/L)	7.35 ± 4.19	5.63 ± 2.86	<0.001
Uric acid (μmol/L)	391.02 ± 115.67	367.95 ± 107.43	0.002
Total cholesterol (mmol/L)	4.50 ± 1.43	4.58 ± 1.24	0.414
Total bilirubin (μmol/L)	13.67 ± 8.53	13.66 ± 7.81	0.983
Glucose (mmol/L)	7.70 ± 3.03	7.07 ± 2.89	0.001
Serum potassium (mmol/L)	4.24 ± 0.49	4.15 ± 0.41	0.003
Serum sodium (mmol/L)	139.35 ± 3.65	139.76 ± 3.03	0.067
Glycated hemoglobin (%)	6.93 ± 1.68	6.72 ± 1.60	0.058
estimated plasma volume status (ePVs)	5.11 ± 1.28	4.03 ± 0.72	<0.001
Neutrophil-to-lymphocyte ratio (NLR)	9.94 ± 7.89	5.80 ± 5.08	<0.001
Left ventricular ejection fraction (%)	50.01 ± 10.44	55.05 ± 7.91	<0.001
N-terminal pro-B-type natriuretic peptide (pg/mL)	11,019.36 ± 10,049.44	3,842.96 ± 2,875.82	<0.001
Haematocrit (%)	0.390 (0.360, 0.426)	0.410 (0.380, 0.442)	<0.001
Peak Troponin-I (ng/mL)	27.58 (5.30,167.71)	20.49 (3.38,100.90)	0.002
Revascularisation (%)	178 (58.4)	1,057 (60.7)	0.238
Ticagrelor, *n* (%)	235 (77.0)	1,333 (76.6)	0.867
Clopidogrel, *n* (%)	154 (50.5)	930 (53.8)	0.339
Aspirin, *n* (%)	304 (99.6)	1,731 (99.4)	0.664
Statin, *n* (%)	304 (99.6)	1,735 (99.7)	0.906
ACEI/ARB, *n* (%)	237 (77.7)	1,367 (78.5)	0.738
CCB, *n* (%)	64 (21.0)	369 (21.2)	0.927
Diuretics, *n* (%)	208 (68.2)	808 (46.4)	<0.001

### Receiver operating characteristic curve analysis

Receiver operating characteristic (ROC) curve analysis was performed to evaluate the predictive performance of ePVS and NLR for cardiovascular mortality. The area under the curve (AUC) of ePVS was 0.794 (95% CI: 0.699–0.887, *P* < 0.001), with an optimal cut-off value of 4.24, a sensitivity of 84.9% and a specificity of 71.3%. The AUC of NLR was 0.736 (95% CI: 0.710–0.763, *P* < 0.001), with an optimal cut-off value of 7.01, a sensitivity of 80.0% and a specificity of 57.6%. These findings indicated that both biomarkers could effectively predict cardiovascular mortality, and ePVS exhibited a better discriminative ability (Figure 1).

**Figure 1:**
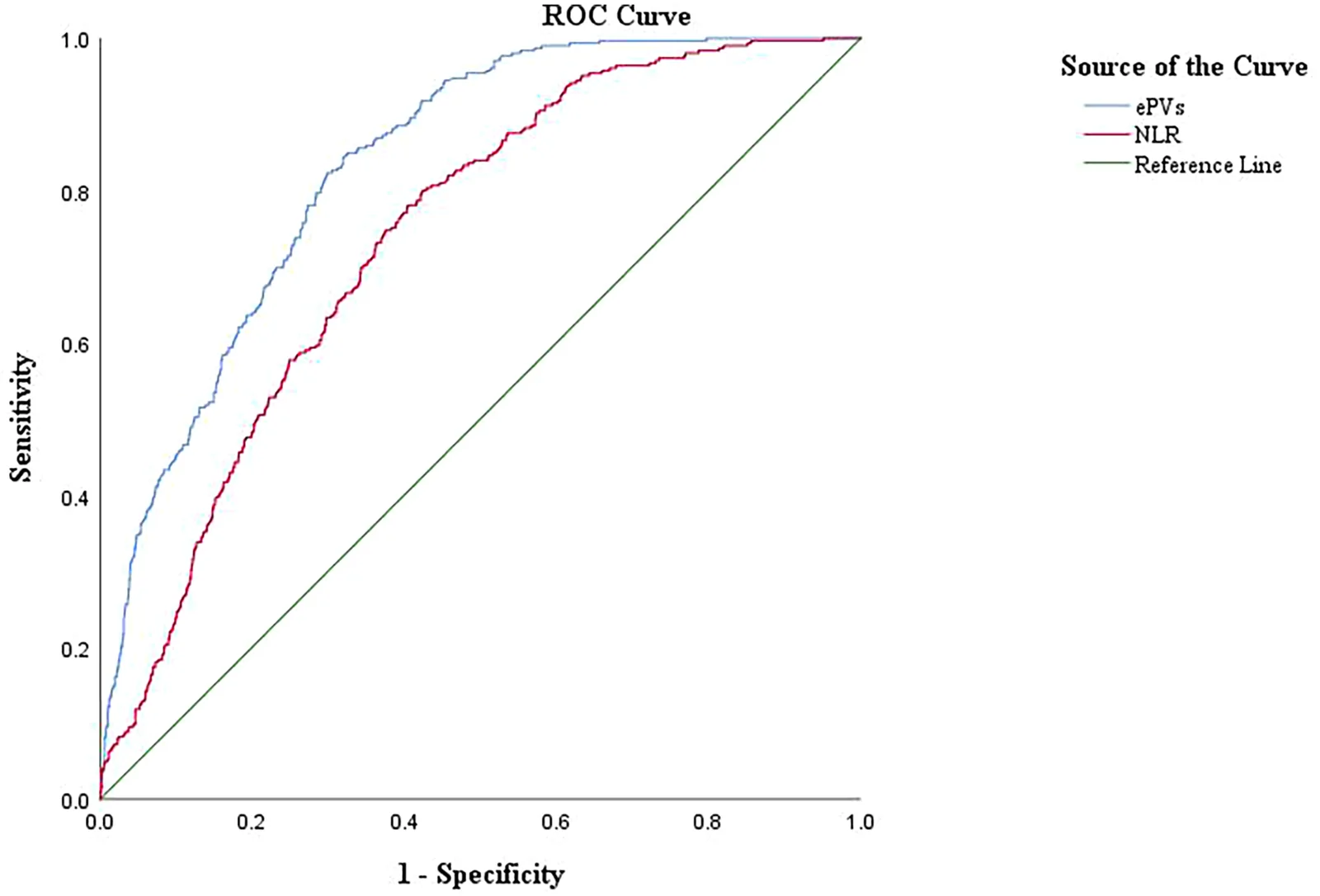
Receiver operating curve showing the area under the curve (AUC) for estimated plasma volume status (ePVs) and neutrophil-to-lymphocyte ratio (NLR).

### Cox proportional hazards regression analysis

Three sequential Cox proportional hazards regression models were constructed to identify predictors of cardiovascular mortality in the study cohort. Univariate analysis (Model 1) showed that elevated ePVS was significantly associated with increased risk of cardiovascular mortality (HR = 1.571, 95% CI 1.502–1.642, *P* < 0.001). In Model 2, after adjustment for age, diabetes mellitus, hypertension, atrial fibrillation, and NLR, ePVS and NLR remained significant predictors of cardiovascular mortality. In Model 3, we further adjusted for STEMI, peak troponin-I, EF, Nt-pro-BNP, BUN, and uric acid. Following full multivariable adjustment, NLR exhibited marked attenuation of its prognostic effect, with the hazard ratio decreasing from 1.330 (95% CI 1.258–1.406) in Model 2 to 1.029 (95% CI 1.017–1.041), although statistical significance persisted. By comparison, ePVS presented moderate attenuation in effect size and retained an independent significant association with cardiovascular mortality. Nt-pro-BNP emerged as a very strong predictor of cardiovascular mortality. Higher EF was associated with lower risk of cardiovascular mortality (Table 2).

**Table 2:** Cox regression analysis for cardiovascular mortality.

Variable	Model 1HR (95% CI)	*P*	Model 2HR (95% CI)	*P*	Model 3HR (95% CI)	*P*
ePVs	1.571 (1.502–1.642)	<0.001	1.436 (1.370–1.505)	<0.001	1.294 (1.194–1.403)	<0.001
NLR	–	–	1.330 (1.258–1.406)	<0.001	1.029 (1.017–1.041)	<0.001
DM	–	–	1.058 (0.830–1.348)	0.649	1.040 (0.802–1.348)	0.767
Hypertension	–	–	1.066 (0.820–1.384)	0.634	0.998 (0.751–1.328)	0.991
AF	–	–	2.309 (1.683–3.169)	<0.001	1.647 (1.152–2.355)	0.006
Age	–	–	1.014 (1.003–1.025)	0.010	1.003 (0.991–1.014)	0.655
STEMI	–	–	–	–	0.920 (0.699–1.210)	0.551
Peak Tn-I	–	–	–	–	1.144 (1.034–1.267)	0.009
EF	–	–	–	–	0.982 (0.968–0.997)	0.015
Nt-pro-BNP	–	–	–	–	7.559 (4.731–12.080)	<0.001
BUN	–	–	–	–	0.965 (0.921–1.012)	0.139
UA	–	–	–	–	1.000 (0.999–1.002)	0.477

Model 1: unadjusted, only ePVS included. Model 2: adjusted for age, diabetes mellitus, hypertension, atrial fibrillation, and NLR. Model 3: adjusted for all variables in Model 2 plus STEMI, peak troponin-I, left-ventricular ejection fraction, Nt-pro-BNP, blood urea nitrogen, and uric acid.

AF, atrial fibrillation; BUN, blood urea nitrogen; CI, confidence interval; DM, diabetes mellitus; ePVs, estimated plasma volume status; HR, hazard ratio; LVEF, left ventricular ejection fraction; NLR, neutrophil-to-lymphocyte ratio; Nt-pro-BNP, N-terminal pro-B-type natriuretic peptide; Tn-I, troponin I; UA, uric acid; STEMI, ST-elevation myocardial infarction.

### Kaplan–Meier survival analysis for cardiac death

The ROC analysis identified 4.24 as the optimal ePVS cut-off for cardiovascular mortality. The ROC analysis yielded an internal optimal cutoff of 4.24 for predicting cardiovascular mortality in the present cohort. However, we adopted the validated cutoff of 4.98 (13) from our previous study as the final threshold for categorical stratification. This externally derived cutoff is clinically consistent with the internal optimal value and facilitates cross-cohort comparison and clinical reproducibility. Patients were stratified into the high ePVS group (ePVS ≥ 4.98, *n* = 70) and the low ePVS group (ePVS < 4.98, *n* = 183). After a 2-year follow-up, the cardiac mortality rate was significantly higher in the high ePVS group than in the low ePVS group (*P* = 0.027) (Table 3). Kaplan–Meier survival analysis yielded consistent results (Figure 2).

**Table 3:** Two-year follow-up results of patients in the high and low estimated plasma volume status (ePVs) groups.

Variables	Cardiovascular mortality group	Survival group
High ePVs (*n* = 275)	130 (47.3)	145 (52.7)
Low ePVs (*n* = 1,771)	175 (9.9)	1,596 (90.1)
*X* ^2^		262.361
*P*-value		<0.001

ePVs, estimated plasma volume status. *n* = 305 events represent cardiovascular mortality only; no composite MACE outcome was evaluated in this study.

**Figure 2:**
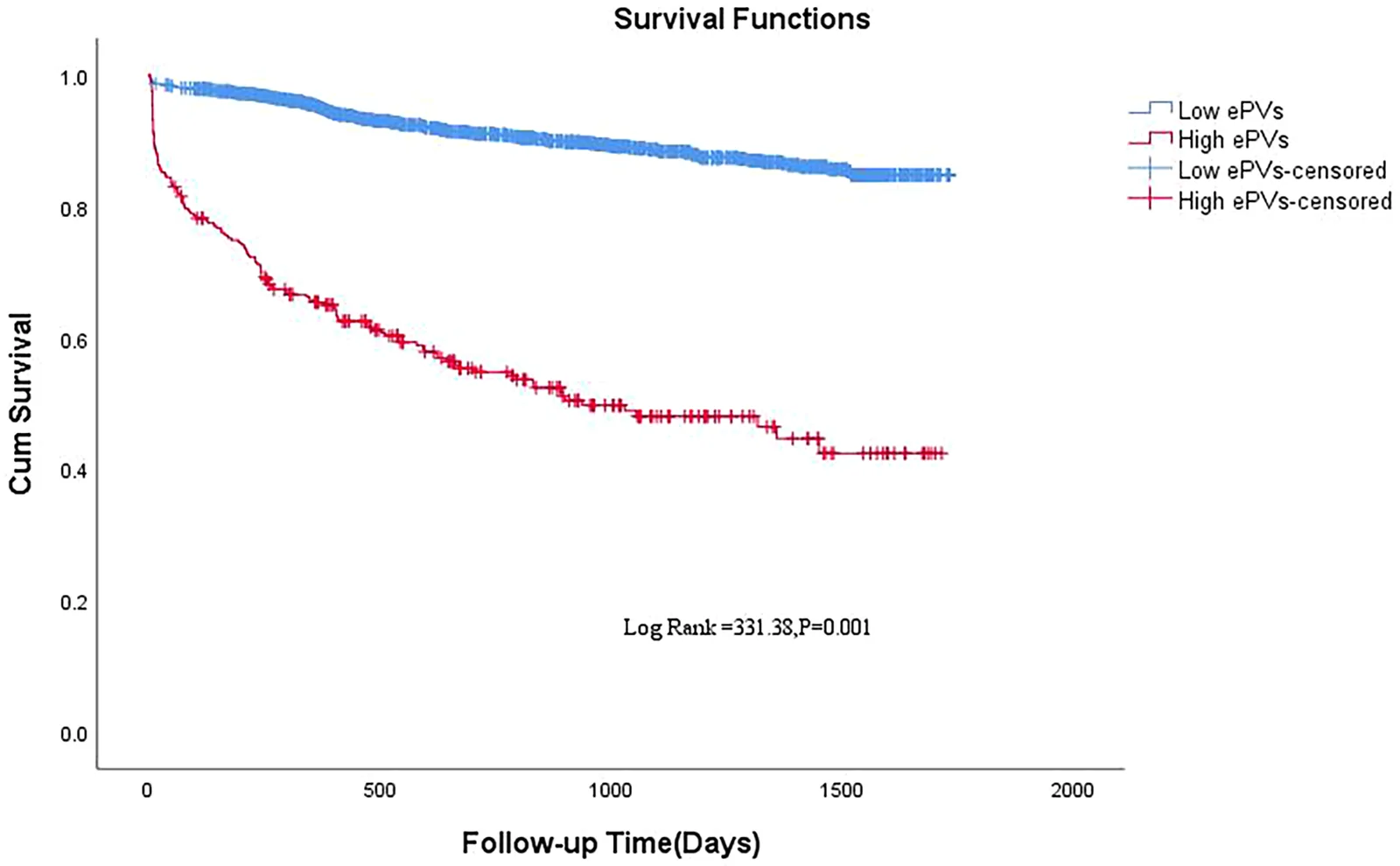
Kaplan–Meier survival curve of 2 year cardiogenic death in patients with high estimated plasma volume status (ePVs) and low ePVs groups.

### Comparison of model performance

We assessed the incremental predictive value of NLR based on the initial model containing ePVS. The AUC increased significantly from 0.794 to 0.891 (*P* = 0.010). Both continuous net reclassification improvement (NRI = 0.948, 95% CI: 0.424–1.471, *P* < 0.001) and integrated discrimination improvement (IDI = 0.100, 95% CI: 0.028–0.172, *P* = 0.006) indicated remarkable improvements in risk reclassification and discriminative capacity. No significant difference was observed in categorical NRI (*P* = 0.798). However, the likelihood ratio test verified that the updated model had a better overall goodness-of-fit (*χ*^2^ = 10.291, *P* = 0.001) (Table 4).

**Table 4:** Comparison of predictive performance between the initial model and updated model for MACE prediction.

Index	Initial model	Updated model	Difference (95% CI)	*P* value
AUC	0.794	0.891	−0.098 (−0.172, −0.023)	0.010
Categorical NRI	–	–	−0.035 (−0.302, 0.233)	0.798
Continuous NRI	–	–	0.948 (0.424, 1.471)	<0.001
IDI	–	–	0.100 (0.028, 0.172)	0.006
Likelihood ratio *χ*²	–	–	10.291	0.001

Exploratory analysis of predictive performance improvement by adding NLR to the single ePVS biomarker model. This unadjusted two-biomarker incremental comparison was performed only to evaluate the combined discriminatory ability of ePVS and NLR. The headline AUC of 0.891 reported in the present study was derived from this unadjusted two-variable model without clinical covariate adjustment. AUC, area under the receiver operating characteristic curve; NRI, net reclassification improvement; IDI, integrated discrimination improvement; CI, confidence interval. Initial model: only ePVs; Updated model: ePVs+NLR.

We further assessed the incremental predictive value of ePVS and NLR on the basis of the pre-specified multivariable clinical model containing age, gender, Killip class, and NT-proBNP, which served as the primary benchmark for incremental evaluation as recommended. The unadjusted dual-marker model combining ePVS and NLR yielded a prominent time-dependent AUC of 0.891, while the fully adjusted clinical model incorporating the two biomarkers achieved improved discriminative performance with a C-index increasing from 0.773 to 0.831 (ΔC-index = 0.059) for 720-day cardiovascular mortality. Both continuous net reclassification improvement (NRI = 0.298, 95% CI: 0.145–0.371, *P* = 0.008) and time-dependent AUC increment (ΔAUC = 0.018, 95% CI: 0.005–0.031, *P* = 0.010) verified significant improvements in risk reclassification and discriminative capacity beyond the established clinical model with dominant predictor NT-proBNP. The integrated discrimination improvement showed a marginal incremental trend (IDI = 0.025, 95% CI: −0.001–0.048, *P* = 0.054). No significant difference was observed in categorical NRI. However, the likelihood ratio test verified that the updated model with ePVS and NLR had a better overall goodness-of-fit (*χ*^2^ = 10.291, *P* = 0.001). Collectively, the addition of NLR and ePVS markedly improved the predictive performance for cardiovascular mortality on the basis of conventional clinical predictors and NT-proBNP (Table 5). Bootstrap internal validation with 500 resamples showed that the original C-index was 0.831 and the optimism-corrected C-index was 0.829, indicating negligible overfitting and stable discriminatory ability. Calibration analysis showed favorable consistency between predicted and observed risks (Supplementary Table S1). DCA further confirmed positive net clinical benefit of the combined biomarker model across clinically relevant threshold ranges (Supplementary Figure S1). Fine-Gray competing-risk regression was performed to eliminate potential bias caused by competing mortality events. After adjustment for age, gender, Killip class, and log-transformed NT-proBNP, both ePVS and NLR remained independent predictors of 720-day cardiovascular mortality. The subdistribution hazard ratios were sHR = 1.239 (95% CI: 1.096–1.40, *P* = 0.00063) for ePVS and sHR = 1.035 (95% CI: 1.015–1.06, *P* = 0.00072) for NLR (Supplementary Table S2). Schoenfeld residual tests demonstrated that the global proportional hazards assumption was generally satisfied. Borderline non-significant trends were observed for age (*P* = 0.050) and ePVS (*P* = 0.057), whereas all other variables fully satisfied the PH assumption, with no substantial model violation (Supplementary Table S3). Pearson correlation analysis showed a weak positive correlation between ePVS and NLR (*r* = 0.101, *P* < 0.001). All VIF values were below 2.1, confirming no evident multicollinearity among covariates.

**Table 5:** Discriminative and incremental prognostic performance of the pre-specified clinical model and the fully adjusted model incorporating ePVS and NLR for 720-day cardiovascular mortality.

Model	Time	Estimate	95%CI	*P*-value
C-index				
NT-proBNP only	720-day	0.736	–	–
Pre-specified clinical model (age, gender, Killip, NT-proBNP)	720-day	0.773	–	–
Clinical model+ePVS+NLR	720-day	0.831	–	–
ΔC-index	720-day	0.059	–	–
Incremental performance (vs. clinical base model, IDI.INF)				
IDI	720-day	0.025	−0.001–0.048	0.054
NRI	720-day	0.298	0.145–0.371	0.008
ΔTime-dependent AUC	720-day	0.018	0.005–0.031	0.010
Single-biomarker time-dependent AUC				
ePVS	720-day	0.845	–	–
NLR	720-day	0.734	–	–

C-index values were calculated based on the full study cohort. Incremental NRI, IDI, and time-dependent AUC improvement were assessed relative to the multivariable clinical base model. Time-dependent AUC of individual biomarkers was calculated among patients with complete biomarker data. The iid-based variance estimator failed to converge for single-marker time-dependent ROC analysis, and only point estimates were presented. Multivariable nested time-dependent AUC comparison was not feasible due to complete data separation.

## Discussion

The present study systematically evaluated and validated the prognostic value of combining ePVS, a biomarker of hemodynamic load, and NLR, an inflammatory and immune marker, for cardiovascular mortality in a large cohort of patients with AMI. To our knowledge, this is the first study to demonstrate that the combined model of ePVS and NLR yields substantially better predictive performance than either biomarker alone. Our results revealed that elevated ePVS was significantly associated with an increased risk of cardiovascular mortality after AMI, which is consistent with findings from previous investigations (14).In a cohort of 1,012 AMI patients, Nogi et al. demonstrated that discharge ePVS > 5.5% was independently associated with increased all-cause mortality (HR = 1.879, 95% CI 1.343–2.629, *P* < 0.001), supporting ePVS as a potential prognostic biomarker in this setting. Another large-scale analysis based on the MIMIC-IV database including 3,238 patients with myocardial infarction complicated by HF further confirmed that patients in the highest ePVS quartile presented elevated risks of in-hospital mortality (OR = 1.58, 95% CI: 1.16–2.13, *P* = 0.003), 180-day mortality (HR = 1.45, 95% CI: 1.19–1.75, *P* < 0.001) and 1-year mortality (HR = 1.51, 95% CI: 1.27–1.80, *P* < 0.001) (15).

AMI remains a leading cause of cardiovascular morbidity and mortality worldwide. Characterized by acute onset, rapid progression and poor long-term outcomes, AMI imposes a substantial clinical and economic burden on public health systems (16). With the widespread application of reperfusion strategies and optimized pharmacological therapies, the in-hospital mortality of patients during the acute phase of AMI has been effectively reduced. Nevertheless, a series of pathophysiological alterations triggered by myocardial ischemic necrosis, including ventricular remodeling, neurohormonal overactivation and inflammatory dysregulation, render HF the most common and severe long-term complication following AMI (17).

The biological rationale underlying the association between ePVS and hemodynamic burden is well established. Impaired left ventricular function after AMI impedes venous return and leads to systemic congestion, and residual congestive status is a critical contributor to adverse post-discharge outcomes in AMI patients. As a practical indicator, ePVS indirectly estimates the degree of plasma volume expansion based on hemoglobin and hematocrit levels, thereby reflecting underlying congestion. Notably, combining ePVS with the GRACE risk score significantly improves the prediction of 5-year mortality in AMI patients. This finding suggests that ePVS possesses promising prognostic implications not only in patients with established HF but also in the general AMI population (18).

NLR is a simple biomarker that reflects the intensity of innate inflammatory responses and the balance of adaptive immunity. An elevated NLR indicates excessive activation of neutrophil-mediated pro-inflammatory reactions alongside impaired lymphocyte-dependent immune repair (19). Accumulating clinical evidence has demonstrated that ischemia-reperfusion injury after AMI triggers a robust inflammatory cascade. Large numbers of neutrophils infiltrate the peri-infarct region and release pro-inflammatory cytokines, oxygen free radicals and proteolytic enzymes, which exacerbate cardiomyocyte necrosis and microvascular damage. A relative reduction in lymphocytes, by contrast, signifies compromised immune defense and reparative capacity, and is closely linked to impaired myocardial scar formation and progressive ventricular remodeling (19). In line with these mechanisms, our study found that patients with a higher NLR had a higher incidence of cardiovascular mortality, and NLR was identified as an independent risk factor for HF development. The optimal NLR cutoff of 7.01 obtained in our cohort is higher than the commonly adopted thresholds of 3–5 in AMI studies (20). This discrepancy may be partly explained by the high-risk profile of our study population and our single endpoint of cardiovascular mortality. It should be noted that this Youden-index-derived threshold yields only moderate specificity (57.6%), as the optimization prioritizes sensitivity for high-risk case detection. This sample-specific cutoff requires further external validation and cannot be directly extrapolated to other AMI populations.

From univariate analysis to fully adjusted multivariate models, both ePVS and NLR independently associated with cardiovascular mortality. Systemic inflammation may correlate with adverse cardiovascular outcomes in this patient cohort, though causal inference cannot be drawn from our observational data. NT-proBNP, troponin and left ventricular ejection fraction, classic robust prognostic indicators reflecting myocardial injury and cardiac function, showed consistent predictive effects in our analysis, which aligns with prior literature. Traditional cardiovascular risk factors including diabetes mellitus and hypertension lost statistical significance after adjustment for inflammatory markers and cardiac function parameters, This observation cannot serve as direct evidence of the superiority of ePVS and NLR. This loss of significance may be due to biological mediators, collinearity, or in the case of the acute myocardial infarction cohort already suffering from the disease, the prognostic performance of traditional risk factors for the disease is generally limited. Additionally, atrial fibrillation persistently increased cardiovascular mortality risk, implying that patients with concomitant arrhythmias have poorer long-term prognosis and require intensified clinical interventions.

Hemodynamic overload and immune-inflammatory responses do not act independently; instead, they interact through complex biological pathways. Volume overload can stimulate the release of pro-inflammatory cytokines by stretching cardiomyocytes and increasing wall stress, thereby amplifying systemic inflammation. Conversely, excessive inflammatory reactions lead to microcirculatory disturbance and increased vascular permeability, which worsen tissue edema and relative insufficiency of effective circulating volume (21). ePVS primarily reflects subclinical volume overload, while NLR mirrors the imbalance between overactivated innate immunity and suppressed adaptive immunity after AMI.

In the present study, we constructed nested models at two levels to comprehensively validate the incremental prognostic value of ePVS and NLR. At the biomarker level, exploratory nested analysis demonstrated that adding NLR to the single ePVS model significantly improved predictive performance, with increased AUC, continuous NRI and IDI, indicating that NLR provides supplementary predictive information beyond ePVS alone. At the clinical model level, which served as the primary benchmark recommended for outcome validation, the incorporation of ePVS and NLR further improved the discriminative ability of the established clinical model consisting of age, gender, Killip class, and NT-proBNP. The C-index increased from 0.773 to 0.831 (ΔC-index = 0.059), accompanied by significant improvements in continuous NRI and time-dependent AUC increment, confirming favorable risk reclassification and predictive gain independent of conventional clinical variables and the dominant predictor NT-proBNP. Of note, IDI exhibited only a marginal incremental trend, and categorical NRI was non-significant. Such discrepancy across different incremental metrics is common in prognostic research, since NRI primarily reflects risk-stratum reclassification, while IDI quantifies overall shifts in individual predicted probabilities. The likelihood ratio test further verified improved model goodness-of-fit after biomarker inclusion. Collectively, ePVS combined with NLR yields incremental prognostic value for cardiovascular mortality, and further external validation is warranted.

The innovation of this study lies in that we are the first to organically combine ePVS and NLR, two inexpensive and readily available biomarkers from routine laboratory tests. Rigorous statistical analyses further validate the incremental prognostic value of this combination. Our findings lay a theoretical foundation for developing a user-friendly risk stratification tool for cardiovascular mortality risk following acute myocardial infarction.

This study has several important limitations. First, this is a single-centre retrospective observational cohort study. We did not perform internal validation and model calibration assessments for the prognostic models. Second, conventional ROC analyses were censoring-naïve and did not account for survival time. Third, optimal cut-off values for ePVS and NLR were derived in a post-hoc manner from the present cohort. Fourth, head-to-head prognostic comparisons against haemoglobin, NT-proBNP and GRACE risk score were not included in the original analysis, which we have now supplemented in revision. In addition, residual confounding cannot be fully excluded despite multivariable adjustment.

## Conclusions

Elevated ePVS and NLR are independently associated with cardiovascular mortality among patients with acute myocardial infarction. The combination of ePVS and NLR yields a better-performing predictive model, with significant improvements in risk reclassification and overall discriminative ability. This combined biomarker strategy may support long-term risk stratification for AMI patients in routine clinical practice. Given the observational nature of this study, direct guidance for individualized therapeutic strategies cannot be inferred from the present findings.

## Data Availability

The raw data supporting the conclusions of this article will be made available by the authors, without undue reservation.

## Supplementary material

Supplementary Figure 1 STROBE flow diagram of patient selection, exclusion, and follow-up outcomes.

Supplementary Figure 2 Decision-curve analysis of the fully-adjusted prognostic model for 720-day cardiovascular mortality. The blue line represents net clinical benefit obtained using model-guided intervention. The red line indicates the strategy of treating all patients, and the green line represents treating no patients. Net benefit fluctuated at high threshold probabilities due to sparse observations in high-risk subgroups.

## References

[1] Harrington J Butler J. Heart failure after myocardial infarction: glass emptier than full. Eur J Heart Fail. (2023) 25(8):1225–7. 10.1002/ejhf.2961 37462280

[2] Bahit MC Kochar A Granger CB. Post-myocardial infarction heart failure. JACC Heart Fail. (2018) 6(3):179–86. 10.1016/j.jchf.2017.09.015 29496021

[3] Harvey PA Leinwand LA. The cell biology of disease: cellular mechanisms of cardiomyopathy. J Cell Biol. (2011) 194(3):355–65. 10.1083/jcb.201101100 21825071 PMC3153638

[4] Sackner-Bernstein JD. The myocardial matrix and the development and progression of ventricular remodeling. Curr Cardiol Rep. (2000) 2(2):112–9. 10.1007/s11886-000-0007-4 10980881

[5] Lin G Li J Huang Y Zhi Y Chen H Chen C. TRIM21 exacerbates hypoxia-induced cardiomyocyte pyroptosis through GSDMD ubiquitination in post-myocardial infarction heart failure. Int Immunopharmacol. (2025) 166:115572. 10.1016/j.intimp.2025.115572 40974637

[6] Frantz S Hundertmark MJ Schulz-Menger J Bengel FM Bauersachs J. Left ventricular remodelling post-myocardial infarction: pathophysiology, imaging, and novel therapies. Eur Heart J. (2022) 43(27):2549–61. 10.1093/eurheartj/ehac223 35511857 PMC9336586

[7] Heusch G. Myocardial ischaemia-reperfusion injury and cardioprotection in perspective. Nat Rev Cardiol. (2020) 17(12):773–89. 10.1038/s41569-020-0403-y 32620851

[8] Jiang H Fang T Cheng Z. Mechanism of heart failure after myocardial infarction. J Int Med Res. (2023) 51(10):3000605231202573. 10.1177/03000605231202573 37818767 PMC10566288

[9] Marinescu MC Lazar AL Marta MM Cozma A Catana C-S. Non-coding RNAs: Prevention, Diagnosis, and treatment in myocardial ischemia-reperfusion injury. Int J Mol Sci. (2022) 23(5):2728. 10.3390/ijms23052728 35269870 PMC8911068

[10] Boulet J Sridhar VS Bouabdallaoui N Tardif J-C White M. Inflammation in heart failure: pathophysiology and therapeutic strategies. Inflamm Res. (2024) 73(5):709–23. 10.1007/s00011-023-01845-6 38546848 PMC11058911

[11] de La Espriella R Palau P Losito M Crisci G Miñana G Domínguez E. Left ventricular volume and maximal functional capacity in heart failure with preserved ejection fraction: size matters. Eur J Heart Fail. (2024) 26(12):2544–52. 10.1002/ejhf.3401 39078308

[12] Lin G Dai C Xu K Wu M. Predictive value of neutrophil to lymphocyte ratio and red cell distribution width on death for ST segment elevation myocardial infarction. Sci Rep. (2021) 11(1):11506. 10.1038/s41598-021-91082-w 34075154 PMC8169857

[13] Chen X Lin G Dai C Xu K. Effect of estimated plasma volume status and left atrial diameter on prognosis of patients with acute heart failure. Front Cardiovasc Med. (2023) 10:1069864. 10.3389/fcvm.2023.1069864 36760561 PMC9905108

[14] Nogi K Ueda T Nogi M Ishihara S Nakada Y Hashimoto Y. Prognostic value of estimated plasma volume status at discharge in acute myocardial infarction. ESC Heart Fail. (2024) 11(5):3222–31. 10.1002/ehf2.14912 39322631 PMC11424303

[15] Luo B Ma Z Zhang G Jiang X Guo C. Estimated plasma volume status as a prognostic indicator in myocardial infarction and heart failure: insights from the MIMIC-IV database. Front Cardiovasc Med. (2025) 12:1499378. 10.3389/fcvm.2025.1499378 40151830 PMC11947685

[16] Kraler S Mueller C Libby P Bhatt DL. Acute coronary syndromes: mechanisms, challenges, and new opportunities. Eur Heart J. (2025) 46(29):2866–89. 10.1093/eurheartj/ehaf289 40358623 PMC12314746

[17] Udell JA Bahit MC Campbell P Butler J Chopra VK Bayés-Genís A. Prevention of heart failure after acute myocardial infarction. Lancet. (2025) 406(10508):1154–70. 10.1016/S0140-6736(25)01394-7 40889512

[18] Kawai T Nakatani D Yamada T Sakata Y Hikoso S Mizuno H. Clinical impact of estimated plasma volume status and its additive effect with the GRACE risk score on in-hospital and long-term mortality for acute myocardial infarction. Int J Cardiol Heart Vasc. (2021) 33:100748. 10.1016/j.ijcha.2021.100748 33748402 PMC7957089

[19] Francisco J Del Re DP. Inflammation in myocardial ischemia/reperfusion injury: underlying mechanisms and therapeutic potential. Antioxidants (Basel). (2023) 12(11):1944. 10.3390/antiox12111944 38001797 PMC10669026

[20] Cao F Jiang J-J Zhang G Liu J Xiao P Tian Y. Prognostic value of inflammatory markers for all-cause mortality in patients with acute myocardial infarction in the coronary care unit: a retrospective study based on MIMIC-IV database. Front Cardiovasc Med. (2025) 12:1439650. 10.3389/fcvm.2025.1439650 39911562 PMC11794309

[21] Ong SB Hernández-Reséndiz S Crespo-Avilan GE Mukhametshina RT Kwek X-Y Cabrera-Fuentes HA. Inflammation following acute myocardial infarction: multiple players, dynamic roles, and novel therapeutic opportunities. Pharmacol Ther. (2018) 186:73–87. 10.1016/j.pharmthera.2018.01.001 29330085 PMC5981007

