# Supplementary material for: Predictive value of combined estimated plasma volume status and neutrophil-to-lymphocyte ratio for cardiovascular mortality in patients with acute myocardial infarction with heart failure
Source: Front Cardiovasc Med. 2026 Sep 28;13:1948520. doi: 10.3389/fcvm.2026.1948520 (PMC13654955; doi:10.3389/fcvm.2026.1948520)
Supplement: Supplementary file 1 [file Table1.docx]

Supplementary Table 1. Model Internal Validation, Calibration and Clinical Net Benefit Evaluation

| Validation Index | Value |
| --- | --- |
| Original C‑index | 0.831 |
| Optimism‑corrected C‑index (500 bootstrap resamples) | 0.829 |
| Calibration intercept | - |
| Calibration slope | - |
| Positive net clinical benefit threshold range | 0%–68% |

Supplementary Table 2. Fine‑Gray Competing‑Risk Regression Analysis

| Variable | sHR | 95% CI | P‑value |
| --- | --- | --- | --- |
| Age | 1.008 | 0.997–1.02 | 0.150 |
| Gender | 0.992 | 0.722–1.37 | 0.960 |
| Killip class | 1.519 | 1.305–1.77 | <0.001 |
| log‑transformed NT‑proBNP | 6.568 | 4.135–10.43 | <0.001 |
| ePVS | 1.239 | 1.096–1.40 | 0.00063 |
| NLR | 1.035 | 1.015–1.06 | 0.00072 |

Supplementary Table 3. Model Assumption Tests and Multicollinearity Analysis

| Parameter | Result |
| --- | --- |
| PH Assumption (Schoenfeld P) |  |
| Age | 0.050 |
| Gender | 0.226 |
| Killip class | <0.001 |
| log‑NTproBNP | 0.037 |
| ePVS | 0.057 |
| NLR | 0.037 |
| Linearity Nonlinearity Test (P) |  |
| ePVS nonlinearity | <0.001 |
| NLR nonlinearity | <0.001 |
| Correlation Analysis |  |
| Pearson r (ePVS vs NLR) | 0.101 |
| Correlation P‑value | <0.001 |
| VIF for All Covariates |  |
| Age | 1.185 |
| Gender | 1.058 |
| Killip class | 1.067 |
| log‑NTproBNP | 2.043 |
| ePVS | 1.894 |
| NLR | 1.134 |
